# The Repetitive Element RLEP Is a Highly Specific Target for Detection of Mycobacterium leprae

**DOI:** 10.1128/JCM.01924-17

**Published:** 2018-02-22

**Authors:** S. Braet, K. Vandelannoote, C. J. Meehan, A. N. Brum Fontes, E. Hasker, P. S. Rosa, N. Lucena-Silva, L. Rigouts, P. N. Suffys, B. C. De Jong

**Affiliations:** a Department of Biomedical Sciences, Institute of Tropical Medicine, Antwerp, Belgium; b Oswaldo Cruz Institute, Rio de Janeiro, Brazil; c Instituto Lauro de Souza Lima, Bauru, Brazil; d Institute Aggeu Magalhães, Recife, Brazil; Carter BloodCare & Baylor University Medical Center

**Keywords:** leprosy, diagnosis, specificity, RLEP qPCR

## LETTER

Leprosy, caused by Mycobacterium leprae, is a mutilating and highly stigmatized disease that still affects hundreds of thousands of new patients annually. The diagnosis relies entirely on clinical findings, per WHO guidelines, although confirmation of clinically doubtful presentations requires reliable diagnostic tools. Early detection and treatment interrupt transmission and prevent severely debilitating disease. Since 2001, the complete genome of M. leprae has been available, which was the basis for several molecular techniques to detect M. leprae (1). Martinez et al. compared four different quantitative real-time PCR (qPCR) assays for leprosy diagnosis using skin biopsy specimens (SBs) from patients (2). They concluded that the qPCR targeting 36 RLEP copies per genome, described by Truman et al. (3), was the most sensitive assay, presenting high sensitivity (100%) for multibacillary (MB; >5 lesions) patients and 84.6% sensitivity for paucibacillary (PB; <5 lesions) patients. Housman et al. tested the RLEP qPCR in both experimentally infected and noninfected armadillos and reported a false positivity rate of 40% (4), raising concerns about test specificity. The specificity might be affected by the presence of homologous sequences in other environmental and understudied Mycobacterium species, which could yield false positives (2). Alternatively, the high sensitivity also makes the assay more prone to contamination as a source of false positives, or the samples tested included true positives in whom leprosy had clinically not been correctly diagnosed, i.e., misclassification of test samples. Thus, our study aimed to revisit the specificity of the RLEP qPCR.

Specificity was first determined *in silico*; the RLEP qPCR primer and probe sequences were compared against the NCBI's nonredundant database using BLASTn (7 December 2017) (5), including 148 sequenced mycobacterial genomes from recent studies (6, 7). This did not identify any potential cross-reactivity. Subsequently, specificity was experimentally tested. Among SBs from 28 and 31 nonleprosy controls tested from areas where leprosy was not endemic or was endemic, respectively, no RLEP qPCR amplification was observed. In addition, none of 61 isolates from different mycobacterial species, including the closely related *M. szulgai* and M. haemophilum, showed amplification for the RLEP qPCR. Confirming sensitivity, all 101 samples from clinically confirmed patients (10 SBs from MB patients and 91 slit skin smears, including 27 acid-fast bacillus [AFB] negative and 64 AFB positive) were positive with the RLEP qPCR. We notified the Institute of Tropical Medicine's Institutional Review Board about testing deidentified surplus diagnostic samples from patients from Brazil, Belgium, and the Comoros, who had provided informed consent.

These results suggest 100% specificity of RLEP qPCR for M. leprae. However, due to the possible presence of homologous RLEP sequences in unidentified, unculturable, or understudied mycobacteria closely related to M. leprae, the reported specificity will always be provisional. The absence of identical primer/probe binding sites in the current NCBI database decreases the probability that new mycobacterial species with homologous RLEP sequences will emerge. Our results suggest that false positives would more likely represent contamination issues. This study supports RLEP qPCR as the gold standard for laboratory confirmation for leprosy, even when sensitivity in PB samples is still imperfect.
